# Bubble collapse dynamics near the composite walls: Progress and challenges^☆^

**DOI:** 10.1016/j.ultsonch.2025.107298

**Published:** 2025-03-10

**Authors:** Yichen Zhu, Xiaojian Ma, Ruiquan Zhou, Yuwei Sun, Mindi Zhang

**Affiliations:** a School of Mechanical Engineering, Beijing Institute of Technology, Beijing 100081, China; b Department of Research and Development, Beijing 100076, China

**Keywords:** Bubble dynamics, Composite wall, Review

## Abstract

Bubble dynamics near the composite walls has become one of the major issues in the fields of aerospace, underwater weapons, and mechanical engineering. The present work reviews recent progress made towards developing experimental and numerical investigation for interaction of bubble dynamics and composite response. The goal of our overall efforts is to (1) summarize the progress made in the experimental and numerical modeling and approaches for bubble dynamics near various composite walls, (2) discuss the effect of designability of the composite materials on the bubble dynamics, with special emphasis on the variations of fiber orientation and ply number of composite walls, as well as correspondingly accompanied by tilted jets and opposite migration of bubbles, with experimental and numerical modeling and approaches, (3) improve the understanding of relationship between bubble dynamic behaviors and material’s specific stiffness via experimental data and modified deep neural network method, with particular emphasis on the critical condition of bubble migration under the actions of various material properties. Issues including the mechanism of bubble–wall interaction are discussed.

## Highlights

• Summarizing the progress made in the experimental and numerical modeling.
• Discussing the effect of designability of the composite materials on the bubble dynamics.
• Improving the understanding of relationship between bubble dynamic behaviors and material’s specific stiffness via modified deep neural network method.

## 1 Introduction

The collapse dynamics of bubble near a wall have been studied for over a hundred years since the basic work presented by Rayleigh. The bubble collapse phenomenon can be observed in many engineering applications. It has been shown that the high-speed jet formed during the final stage of bubble collapse and the bubble migration both direct towards a rigid wall [1], [2], [3], which are major factors causing structural damage [4], [5], [6], vibration [7], [8], [9], and noise [10], [11].

The bubble collapse process can be characterized by the bubble shapes, migration, jet, collapse time, and shock wave, and so on [12]. The interaction between bubble and neighboring wall play important role in the process of bubble collapse dynamics. The bubble behaviors, including migration and final jet formation are influenced by the properties of neighboring wall. For example, the bubble will be attracted by the rigid wall with the direction of the final jet towards the rigid during the later stage of collapse [13], and repelled by the free surface with the direction of final jet away from the surface [14]. When in close proximity to the flexible wall, the bubble collapse behaviors are between the above two situations. If the material properties of the walls are changed, the bubble collapse dynamics will be changed according the interaction of bubble flow and wall structure, which is not only an interesting topic, but also has potential applications in engineering.

Advanced fiber-reinforced resin–matrix composites with high performance reinforcements have been widely used in aerospace, rail transit, marine engineering and civil engineering [15] due to their high specific strength, high specific modulus, high temperature resistance, corrosion resistance, fatigue resistance, and designable performance [16]. The great difference between composite materials and metals lies in the designability of materials [17], [18]. The performance of fiber reinforced resin matrix composite depends not only on the performance of fiber and matrix itself, but also on the microstructure designability, including fiber content and fiber angle, ply angle, ply sequence and ply thickness, etc. [19], [20]. In order to change the macro mechanical properties of the materials (i.e. specific stiffness), the micro structure can be designed and optimized according to the load conditions and the form of the structural components. In the other word, the fiber in the composite material can be designed into the appropriate content and laid reasonably [21], [22]. In addition, due to the self-adaptive bending-twisting coupling deformation of fiber-reinforced resin–matrix composite under the action of bubble behaviors, the impact load from bubble collapse to the structure can be weakened significantly [23].

To carry out in-depth basic research around this issue of the bubble behaviors interacted with carbon fiber reinforced polymer (CFRP) composites can provide basic theories and methods for solving the major engineering needs. It could also enhance the integration of multi-disciplines, and effectively promote the overall improvement of the research quality.

### 1.1 The key aspect in bubble dynamics behaviors near various wallsats

#### 1.1.1 Experimental study

Review of the literature regarding the investigation method, boundary types, and typical bubble properties in experimental study is summarized in Table 1 to facilitate the understanding of the mechanism of bubble behaviors, and also for comparing with the simulation results.

Various experimental techniques have been developed to study the bubble dynamics near various walls. A combination of visualization system and bubble generator is widely applied in the research of bubble dynamics. According to different research purposes and conditions, there are three ways to generate bubbles, namely, underwater explosion bubble [24], laser-induced bubble [25], [26], and electric-spark bubble [27], [28]. A bubble generation method for low voltage discharge was developed by Zhang et al. [31] based on electric-spark bubble method, which has been widely used because of its cheap equipment, convenient operation and good controllability. As for the visualization system, the high-speed camera is widely used to record the temporal evolution of bubble shapes [32], [33], [34], [35], [36], while the shadowgraph/schlieren imaging is adopted to capture the propagation of shock waves and rarefaction waves [37], [38], [39].

**Table 1:** Summary of experiments for bubble dynamics.

References	Investigation method	Boundary types	Conclusions
Hung et al. [24]	High-speed camera Underwater explosion	Metal	Stiffness, inertia, and phase between plate vibration and the bubble pulsation are important parameters to affect the bubble migration.
Brujan et al. [25], [26]	High-speed camera Laser-induced bubble	Polyacrylamide (PAA)	The elastic modulus of PAA is important factor to affect the bubble migration.
Huang et al. [27]	Shadowgraph imaging Electric-spark bubble	Between rigid wall and free surface	Shock wave and rarefaction wave are observed to affect the bubble collapsing behaviors.
Zhang et al. [28]	High-speed camera Electric-spark bubble	Rigid boundary, free surface	Resultant force of buoyancy force and Bjerknes force determines the bubble migration.
Reuter et al. [29]	High-speed camera Laser-induced bubble	Rigid boundary	The formation mechanisms behind the needle-jet and the regular jet are very distinct.
Zhang et al. [30]	High-speed camera Laser-induced bubble	Rigid boundary	The counter-jet generated in the rebound stage will drive the cavitation bubble to move away from the wall.

In particular, microjets emitted during bubble non-spherical collapse have been regarded as primarily responsible for the generation of the high and impulsive pressure, resulting in destruction load related to fluids engineering [32], [33], [34], [35], [37]. Here, ‘jets’ always refers to the following process: one of the bubble margins moves inwards and pierces the bubble interior before impacting the opposite side of the bubble margin. Microjets have the diverse origins, including rigid boundaries or free surfaces [36], [40]. Brujan et al. [25], [26] observed that bubbles near an elastic boundary can be mushroom-shaped and split into two smaller sub-bubbles. The response of an elastic boundary is believed to provide additional energy to a bubble and generate an annular jet around the bubble surface, eventually resulting in two axial jets in opposite directions. Their temporal evolution strongly also depends on the external factors, especially for the gravity [41], [42], shock waves [43], [44], acoustic waves [45], [46], and the properties of the surrounding liquid [47], [48], [49].

Bubble migration is another important feature significantly influenced by the boundary type. Extensive studies on the dynamic behaviors of bubble migration near different boundaries have been reported by Hung et al. [24], Brujan et al. [25], [26], Klaseboer & Khoo [42], and Blake et al. [50]. Conclusions can be summarized as that a bubble follows different migration directions near various boundaries; for example, bubbles migrate towards a rigid boundary and away from a free surface. If a boundary possesses evident elastic properties, a “neutral collapsing bubble” can occur, which travels neither towards nor away from a polyacrylamide (PAA) boundary, according to the experimental observations reported by Brujan et al. [25], [26]. A similar behavior was also observed for bubbles generated near a flexible material coated on a solid surface reported by Gibson & Blake [51] and Plesset & Prosperetti [52]. In addition, bubble migration near a fluid–fluid surface was investigated by Klaseboer & Khoo [42]. They found that the direction of bubble jet motion depends on the density ratio of the two fluids.

The bubble migration is closely relate to the dynamic response of boundary. Hung et al. [24] experimentally investigated the relationship between a bubble generated in an underwater explosion bubble and the boundary response. They found that the phase between the boundary vibration and bubble pulsation may be an effective index for predicting bubble migration. However, more systematic works regarding the underlying mechanism and relationship between those two parameters should be conducted. For example, the effect of natural frequency should be paid more attentions on bubble migration, which is a function of the inertia and stiffness and may provide a reasonable explanation for bubble migration.

The material properties also make the significantly influence on the bubble migration. Gibson & Blake [51] introduced the effects of the boundary inertia and stiffness on bubble migration near composite rubber coatings with different thicknesses. They found that bubble migration and the corresponding behaviors were clearly influenced by the effects of these two parameters. Moreover, Shima et al. [53] performed a more detailed set of experiments on spark-generated bubbles near a composite structure. They found that the bubble moves towards the composite when the inertia coefficient of the composite is large, while the bubble moves away from the composite when the inertia coefficient is small. They also showed that the direction of bubble migration depends not only on the natural properties of a boundary, but also on the bubble size and initial standoff. Duncan et al. [54] and Tomita & Kodama [55] also investigated the collapse of a bubble near a compliant boundary, taking into account the inertia and stiffness constant of the walls.

#### 1.1.2 Numerical modeling

Phenomenologically, bubble collapse dynamics is a complex and multiphase flow phenomenon, and the physical mechanisms have not been fully understood because of the significant morphological variations of bubbles and different compressibility of gas and liquid, etc. Hence, there are significant challenges for the computationally simulation of bubble collapse dynamics, such as the accuracy, stability, and efficiency of those numerical methods.

The commonly numerical methods of simulating bubble collapse dynamics include potential flow theory and viscous flow theory summarized in Table 2. The boundary element method (BEM) is the most representative of the potential flow theories to simulate the bubble collapse dynamics, whose basic idea is to transform the differential equation into the integral equation on the gas–liquid interface through the Green formula. BEM has two important advantages in simulating the bubble collapse dynamics: (i) Green function transforms the three-dimensional potential flow theoretical equation describing the bubble movement into two-dimensional boundary integral equation, so that the calculation amount can be saved by reducing by one dimension; (ii) The gas–liquid interface coordinates can be solved directly by using the BEM, and it is not necessary to apply the front-tracing method to capture the interface between gas and liquid.

However, the BEM still has its natural limits in accurately simulating the transient evolution of bubbles. Firstly, it is impossible to use Green’s formula to reduce the order of higher-order viscosity term, so the BEM is generally applicable to the numerical simulation of inviscid flow. Furthermore, BEM has met some difficulties to accurately simulate the splitting behaviors in rebound stage of bubbles without the application of interface-tracing methods, due to the changes of calculation domain from simplify-connected domain to multiply-connected domain. In order to overcome those defects, BEM made a significant progress in considering the viscosity term [60] and multiply-connected domain [61], which greatly improve the simulation accuracy of the bubble collapse dynamics.

**Table 2:** Summary of numerical models for simulation of bubble dynamics.

References	Investigation method	Conclusions
Guo et al. [56]	Potential flow theory (Boundary-element method, BEM)	Lack of the ability to perform the viscosity term.
Duncan et al. [54]	Potential flow theory (BEM)	Lack of the ability to simulate the toroidal bubble shape in rebound stage.
Turangan et al. [57]	Potential flow theory (BEM)	Displayed the compressibility of liquid.
Koukouvinis et al. [58]	Viscous flow theory (Navier–Stokes (N–S) equation)	The accuracy of the model and the numerical results are well agreed with experiment data.
Supponen et al. [48]	Viscous flow theory (N–S equation)	Agreed well with the experimental result.
Lauer et al. [59]	Viscous flow theory (N–S equation)	The compressible treatment could improve the dynamics description.

The viscous flow theory has gradually become the main method to study the bubble collapse dynamic behaviors due to its excellent performance of simulating viscosity effect and rebounded bubble shapes. Its main idea is to solve the Navier–Strokes (N–S) equation, as well as the two-phase flow model (i.e. phase function [62]) and the front-tracing method (i.e. VOF [63] and LS [64]) to realize the accurate simulation of the bubble pulsation process. The works by Tian et al. [65], Liu et al. [66], Koukouvinis et al. [58], Supponen et al. [48], and Lauer et al. [59] simulated the bubble dynamics by solving the N–S equation. The results show that the accuracy of the model and the numerical results are well agreed with experiment data.

However, due to the lack of pressure term in the mass conservation equation, it is necessary to solve the pressure Poisson equation or pressure correction method in N–S equation, which greatly wastes the calculation costs. Therefore, in the framework of compressible N–S equation, Caltagirone et al. [67] constructed a specific equation in function of the velocity and pressure by solving the continuity equation and the state equation together to get the multi-order formula of pressure on Mach number, which saves significant calculation resources. The research results show that the method can effectively predict the strongly compressible bubble behaviors and weakly compressible fluid structures.

Accurate physical model is essential for the efficient simulation of bubble collapse dynamics, but the post-processing method also plays an important role in the in-depth analysis of numerical results. The complexity of the evolution process of pressure wave system under the action of bubble pulsation poses a significant technical challenge to the numerical work of pressure wave system identification. It is known by the works of Koukouvinis et al. [58] that bubble could emit the complex and various pressure waves during pulsation, such as temporal evolution of rarefaction wave and shock wave, as well as their interaction.

However, the traditional identification methods or post-processing methods of pressure waves, such as pressure contour and Mach number contour, cannot accurately and quantitatively distinguish those various types of pressure waves induced by the bubble pulsation, due to three main points: (i) The evolution of pressure wave under the action of bubble pulsation is a complex physical process with strong nonlinearity and strong instantaneity, including the dynamic behaviors of compression and rarefaction waves, as well as their interaction, interference and transformation on the time scale [68]; (ii) The interaction of the complex pressure waves and the unsteady pulsating bubble multiphase flow, as well as the coupling of the structural boundary, will produce the secondary flow structures, which further complicates the flow fields and the pressure wave fields on the spatial scale [69]; (iii) The dissipation and oscillation of numerical results will smooth the discontinuities in the flow field, resulting in that the weak wave (rarefaction wave) cannot be recognized [70].

In their work, Johnsen & Colonius [71] applied the numerical Schlieren contours to descript the propagation of various pressure waves. The results showed that it is appropriate for capturing variously different pressure waves. Furthermore, the acoustic impedance theory introduced by Gabard et al. [72] is applied in distinguishing the development and transformation of various pressure waves. The results show that acoustic impendence theory better capture the transformation between different pressure waves with distinct physical characteristics.

The open-source CFD code OpenFOAM is used for the numerical simulation of the experimental case, and more details of the flow field in the collapse process of the near-wall cavitation bubbles are obtained. Zhang et al. [30] simulated the collapse process of the cavitation bubble near the solid wall with the help of OpenFOAM code. It is found that the numerical simulation of the cavitation bubble morphology in the collapse stage is well consistent with the experimental results and the obtained micro-jet velocity of the cavitation bubble collapse can reach 170 m/s, which is in the same order of magnitude as the experimental micro-jet velocity. And the obtained velocity of the pressure wave is only the order of the underwater sound velocity in the numerical simulation. However, a clear shock wavefront is not obtained, the ability to capture the shock wavefront needs to be improved in our future research.

### 1.2 The key aspect in modeling of advanced composite materials

Material is the foundation and leading technology of modern new technology development. Recently, fiber reinforced polymer matrix composites are widely used in engineering because of their good specific stiffness, specific strength, and designability, as well as excellent hydrodynamic characteristics [73]. The commonly used types of reinforcing fibers include carbon fiber and glass fiber [74].

In the field of marine and aerospace equipment with increasingly severe loading conditions, it is an important prerequisite to establish the theory and technology, which can accurately predict the mechanical properties of composite materials. The structural analysis of composite materials involves two scales: one is the macro-scale with the average value [75]; the other is the micro-scale, which involves the properties of component materials and the distribution of microstructure [76].

Because the micromechanical analysis method is based on the concept of particles under the framework of discontinuous medium mechanics, it has the disadvantages of complicated calculation and process, generally not used in the process of interdisciplinary research [77], [78]. In the contrast, the macro mechanical analysis method of composite material is a kind of mechanical analysis method, which regards composite material as macro homogeneous medium from the view of phenomenology. A large number of researchers, such as Ju et al. [79], Huang & Zhang [80], Limouei et al. [81], Kramer et al. [82], and Grover et al. [83], have studied the mechanical properties of the composite materials based on the macro-mechanical method, which provides an efficient theoretical model for the engineering application of composite materials.

In macro mechanical analysis method, the reinforcement phase and the matrix are considered as an entirety, without considering the interaction between the components of composite materials, only considering its macro and average mechanical properties [84]. Furthermore, the mechanical parameters such as stress and strain in the macro-mechanical method are defined not the real stress and strain of matrix phase and reinforcement phase, but just the average value on the macro scale. According to the homogeneous anisotropic materials, the macroscopically mechanical method usually establishes the constitutive model of composite materials, and necessarily mechanical parameters used to predict the response of the composite materials are obtained from macroscopic experiments [85]. The macro-structure analysis of composite materials in the elastic range is carried out by anisotropic elastic mechanics or finite element method, and the method is relatively mature.

### 1.3 The key aspect in interaction of bubble and composite wall

As mentioned in the above research, the high-speed jet and shock wave emitted by bubble collapse are important factors to cause destruction load for fluids engineering structure. The advanced composite is becoming a primary concern for solving the devasting consequences of the bubble behaviors. The work by Shima et al. [53] experimentally measured the unsteady bubble behaviors near the composite surface consisting of rubber and foam. They considered that bubble migration depends on the properties of composite surface (i.e. stiffness and inertia), bubble size, and initial distance. Also, Tomita & Kodama [55] analyzed bubble migration near the rigid/rubber composite plate glued on a foam rubber and showed that the bubble migration was significantly influenced by the dynamic response of composite surfaces. The study by Gong et al. [86] investigated the interaction of bubble and two-layered composite beam consisting of metal and foam rubber. And they found bubble collapse time is greatly influenced by the nearby two-layered composite beam. Although the interaction between various composite materials and bubbles have attracted much attentions in the previous works, researchers just focused on the isotropic medium of solid, but not referred to the advanced fiber reinforced resin composite with anisotropic ability. Furthermore, the Fluid–Structure Interaction (FSI) provides a technology challenge in the fluid–structure interface when carrying out the simulations.

Recently, the development of the numerical technique has resulted in noticeable efforts made to use numerical simulation tools. An overview of previous FSI model study for simulating bubble behaviors near composite walls, which considers the solution of both the fluid and structure domain, is given in Table 3. The FSI models of fluid and structure domain in the field of bubble dynamics near composite walls can be generally divided into three categories: doubly asymptotic approximation (DAA), modified ghost-fluid method, and specific BEM/FEM coupling model.

Mccoy and Sun [87] proposed to use the DAA for modeling the surface interaction of a structure, submerged in an infinite fluid medium. DAAs are differential equations used in the analysis of a flexible structure immersed in an infinite fluid medium, which is based on representing the surface motion as a linear combination of orthogonal fluid boundary modes.

**Table 3:** Summary of FSI models for simulation of bubble dynamics near composite walls.

References	FSI model	Objectives
Mccoy & Sun [87]	Doubly asymptotic approximation	FSI analysis of a thick-section composite cylinder subjected to underwater blast loading.
Young et al. [88]	Modified ghost-fluid method	FSI effects of underwater explosions and composite structures.
Ge et al. [68]	Modified ghost-fluid method	Investigation of underwater explosion near composite structures.
Gong et al. [89], [90]	Fully 3D BEM/FEM coupling model	The dynamic response of the two-layered composite beam induced by the bubble growth and collapse.
Hsiao [91] & Chahine	BEM/FEM coupling model	Dynamic response of a composite propeller blade subjected to shock and bubble pressure loading.

Ge et al. [68], Young et al. [88], and Liu et al. [92] applied the modified ghost-fluid method (MGFM) to achieve the interaction between the composite and bubble. The MGFM is reformulated for fluid–solid coupling by treating simultaneously the fluid characteristics equation and the solid equation of motion to determine the interface variables, leading to a strongly coupled fluid and solid scheme.

The studies reported by Gong et al. [89], [90] and Hsiao & Chahine [91] achieved the FSI effect of composite propeller blade and bubble by a fully BEM/FEM coupling model at each time step the corresponding fluid code and the structure code. Klaseboer et al. [42] introduced its detail steps of FSI model as follows: The pressure loading on the nodes in the fluid are calculated using the BEM solver and then transferred to the FEM solver; The FEM solver then calculates the displacements of the two-layered composite beam to be as inputs in the fluid BEM solver. And finally, the cycle continues.

However, all the above FSI models can be summed up as body-fitted grid technology. There are many defects in dealing with the coupling problem of fluid and complex structure boundary, such as the difficulty of high-quality grid generation, the large deformation of grid, etc., which brings great difficulties to the construction of high-precision grid with strong robustness.

The new research direction of FSI algorithm is immersed boundary method (IBM), one of non-body fitted grid methods, to achieve the data interchange between flow field and structure field at the interface. The main idea of the IBM is to represent the effect of the dynamic response of the structure on the fluid by modeling the complex structure into the force source term of the momentum equation of the N–S equation.

Wang et al. [93], [94] proposed a two-dimensional IBM for the coupling of large deformation and compressible multiphase flow. In the framework of IBM, Schwarz et al. [10] proposed a numerical method for simulating the variable shape of interface by spherical harmonic series expansion method. The calculated curvature error of the surface is much smaller than that of the surface represented by discrete mesh points.

### 1.4 Scopes

Although much attention has been paid to the bubble dynamics near various walls in recent decades, the interaction between bubble behaviors and anisotropic composite walls makes the dynamic characteristics of bubbles and composite walls even more complicated. The objectives of this article are to review the classic experiment tools to obtain comprehensive data of bubble behaviors, and the computational tools for interaction between bubble dynamics and anisotropic composite wall. The emphasis of the simulation is on the updated compressible flow model, and anisotropic structural equation based on macro mechanics, along with the FSI model based on IBM model. The present review will:

(1) Summarize the progress made in the experimental and modeling study in the bubble collapse behaviors near the anisotropic composite walls.
(2) Discuss the effect of designability of the composite materials on the bubble dynamics, with special emphasis on the variations of fiber orientation and ply number of composite walls, as well as correspondingly accompanied by tilted jets and opposite migration of bubbles.
(3) Improve the understanding of relationship between bubble behaviors and material’s specific stiffness, with particular emphasis on the critical condition of bubble migration under the actions of various material properties.
(4) Propose current challenges and propose future research directions in the study flied.

## 2 Experimental setup and method

In order to study the interaction of bubble collapse and composite materials, a synchronous measurement platform of bubble shapes and structural response is established, focusing on the Joule heating bubble generator and composite walls.

### 2.1 Synchronous measurement platform of bubble shapes and structural response

**Fig. 1:**
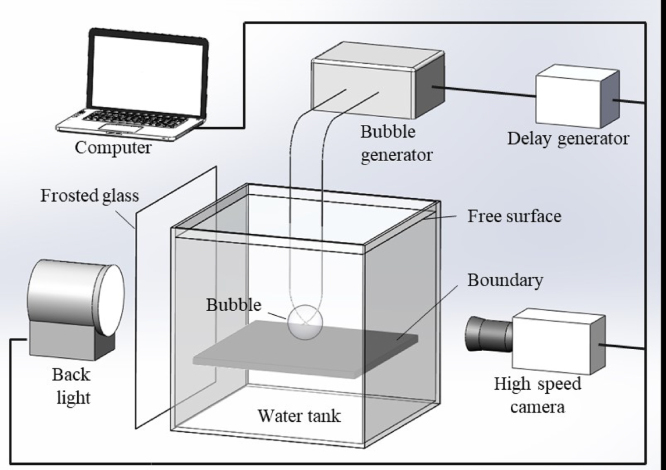
Synchronous measurement platform for the interaction between bubble shapes and structural response [96].

Fig. 1 shows the schematic diagram of the synchronous acquisition system [95] of bubble shapes and structural response. The system includes high-speed camera, light source, electric-spark bubble generator, water tank and computer processing system. The experimental methods mentioned in Section 1.1 are applied in this system, except for the way of generating bubbles, which uses only electric-sparking generation. An electric-spark bubble generator is applied to incept the single bubble at the connecting of two copper electrodes. The advantage of this approach is that the bubbles generated are larger in size and have a greater impact on the composite wall, which is more conducive to the study of the effect of bubbles on the composite wall at this scale, and therefore the following studies all use this bubble generation approach. A piece of frosted glass is placed between the light source and the single bubble to obtain the uniform light distribution around the bubbles. The high-speed camera is adopted to collect the transient shapes of bubbles and the dynamic deformation of composite walls. The images of bubbles and the composite walls are processed and restored in the computer. A delay generator is used to control the trigger signals of high-speed camera, bubble generator, computer, and light source to achieve the synchronous measurement.

### 2.2 Parameters of single bubble used in the study

In present work, bubbles are generated via low-voltage discharge at the connecting point of the electrodes resulting from the discharge of a 6600 μF charge to 800 V [97], [98]. Upon discharge, copper electrodes, with a 0.25 mm diameter, evaporate the water at the connecting point, emit an extremely high temperature, and create a bubble with rapid expansion, namely, a spark-induced bubble. To quantitatively describe a bubble in an infinite fluid, Shima et al. [53] proposed a parameter, the maximum radius of the bubble, which is defined as

(1) $$R_{m}=\sqrt{A/\pi }$$

where A is the maximum area of the bubble on the screen. The center of the initial bubble is shown to always be located at the connecting point. Therefore, it is possible to precisely control the spatial location of the initial bubble. In the present experiment, bubbles are generated over the boundary. The dimensionless standoff distance between a bubble and the boundary is defined as

(2) $$\gamma =\frac{L}{R_{m}}$$

where L is the distance from the bubble center to the boundary at inception.

### 2.3 Parameters of composite plates used in the study

CFRP composite material is used as the target boundaries in present work. In order to investigate the effect of ply number and the fiber orientation on bubble dynamics, two different groups of composite walls are investigated.

**Fig. 2:**
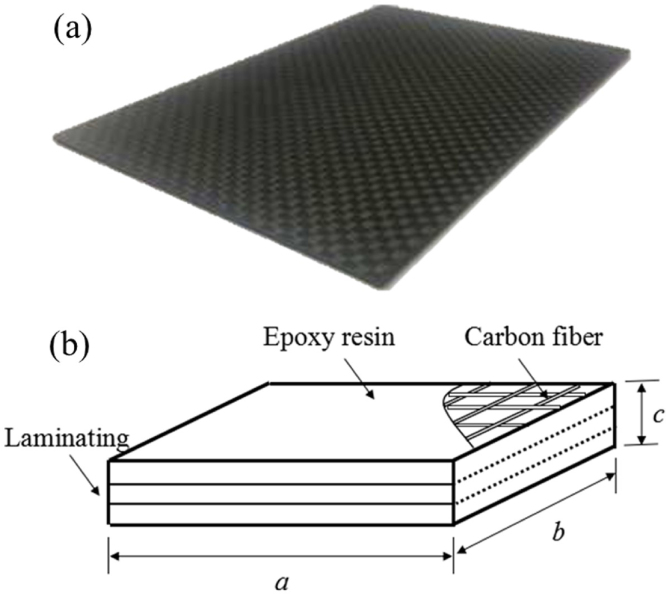
Physical drawing and schematic diagram of composite fiber layer design [96].

The first group is that CFRP composite walls have different ply numbers. As shown in Fig. 2, in order to study the dynamic response of the composite under different fiber layers, the composite walls are machined into different fiber ply number with n= 15, 10, 6 and 3, respectively. Four pieces of composite plates are cut into rectangular plates with a= 120.0 mm and b= 75.0 mm, but different thickness c. Each carbon fiber layer is cross braided and hence its macroscopic mechanical properties are isotropic with Young’s modulus E= 66.2 GPa and Poisson’s ratio v= 0.43. And the detail information can be found in Table 4.

**Fig. 3:**
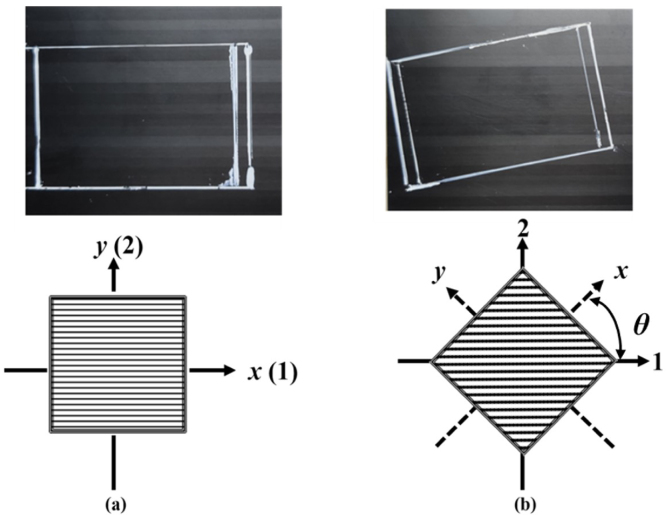
Physical drawing and schematic diagram of composite materials with different fiber orientations [96].

The second group is that CFRP composite walls have different fiber orientations. An anisotropic carbon fiber composite board with n= 8 is treated as the target boundary, which are also composed of carbon fibers and epoxy resin. As shown in Fig. 3, two different fiber orientations are investigated in this paper, namely, θ= 0°and θ= 30°. This kind of anisotropic composite exhibits differently mechanical properties along the fiber direction and perpendicular to the fiber direction, as shown in Table 5. Furthermore, the composite board is cut into rectangular plates with a length of a= 120 mm, a width of b= 75 mm and a thickness of c= 1.5 mm.

**Table 4:** Detailed parameters of composite plates with different ply numbers.

Layer number n	15	10	6	3
Length a/mm	120.0	120.0	120.0	120.0
Width b/mm	75.0	75.0	75.0	75.0
Thickness c/mm	3.0	2.0	1.0	0.5
Mass M/g	48.0	33.1	15.1	6.2
Stiffness k/N/mm	310.1	91.9	11.5	1.4

**Table 5:** Detailed parameters of carbon fiber.

Along fiber direction		Perpendicular to fiber direction		Shear modulus
$E_{1}$/GPa	$v_{12}$	$E_{2}$/GPa	$v_{21}$	$G_{12}$/GPa
42.83	0.305	6.48	0.045	5.48

## 3 Modeling and numerical approaches

The FSI simulation algorithm is proposed to calculate the interaction of bubble dynamics and anisotropic composite materials, including the modified compressible Naiver–Stokes equation, the anisotropic vibration equation of composite wall, and the IBM to achieve the multi-field coupling calculation.

### 3.1 Governing equation of fluid domain and identification of pressure waves

Caltagirone et al. [67] proposed a modified N–S equation to simultaneously manage strongly and weakly compressible fluid motions within the framework of single fluid model. The governing equation system includes

(3) $$\left\{\begin{array}{c} \frac{\partial p}{\partial t}+\frac{1}{\chi_{T}}\nabla \cdot u=0\\ \overset{\sim }{\rho }\left(\frac{\partial u}{\partial t}+\left(u\cdot \nabla \right)u\right)\\ \;=\overset{\sim }{\rho }g-\nabla \left(p-\frac{\tau }{\chi_{T}}\nabla \cdot u\right)+\nabla \left(\overset{\sim }{\mu }\left(\nabla u+\nabla^{T}u\right)\right)+F_{TS}\\ \frac{\partial C}{\partial t}+u\cdot \nabla C=0 \end{array}\right)$$

where p is the pressure; t is the time; u is the velocity; g is the gravitational acceleration; $\chi_{T}=\frac{1}{\rho }\frac{\partial \rho }{\partial p}$ is the adiabatic compressibility; τ is a characteristic time of compressible effects linked to inertial time; $F_{TS}$ is the surface tension force. The density and viscosity of the two-phase fluid are typically defined as $\overset{\sim }{\rho }=C\rho_{l}+\left(1-C\right)\rho_{g}$ and $\overset{\sim }{\mu }=Cu_{l}+\left(1-C\right)u_{g}$, where subscripts l and g denote the liquid phase and gas phase, respectively. C is the local volume fraction, 1 for liquid phase and 0 for gas phase. VOF method is used to track the gas–liquid interface.

### 3.2 Governing equation of composite structure domain

Shen and Hu [99] proposed a differential equation of anisotropic vibration of rectangular thin plates, which is defined as

(4) $$D_{11}\frac{\partial^{4}\omega }{\partial x^{4}}+2\left(D_{12}+2D_{66}\right)\frac{\partial^{4}\omega }{\partial x^{2}\partial y^{2}}+D_{22}\frac{\partial^{4}\omega }{\partial y^{4}}+4D_{16}\frac{\partial^{4}\omega }{\partial x^{3}\partial y}+\;4D_{26}\frac{\partial^{4}\omega }{\partial x\partial y^{3}}+\rho \frac{\partial^{2}\omega }{\partial t^{2}}=q$$

where ω is the deflection (or displacement) in z-axis; q is the external loading; ω is the function of x and y, namely ω=ω(x,y); and $D_{ij}$ is the stiffness coefficient of internal moment related to curvature and twist rate, collectively called bending stiffness and defined as

(5) $$D_{ij}=\frac{1}{3}\overset{n}{\underset{k=1}{\sum }}\left({\overset{¯}{Q}}_{ij}\right)\left(z_{k}^{3}-z_{k-1}^{3}\right)$$

where n is the ply number of fibers, and

(6) $${\overset{¯}{Q}}_{11}=Q_{11}\cos^{4}\theta +2\left(Q_{12}+2Q_{66}\right)\sin^{2}\theta \cos^{2}\theta +Q_{22}\sin^{4}\theta$$

$${\overset{¯}{Q}}_{12}=\left(Q_{11}+Q_{12}-4Q_{66}\right)\sin^{2}\theta \cos^{2}\theta +Q_{12}\left(\sin^{4}\theta +\cos^{4}\theta \right)$$

$${\overset{¯}{Q}}_{22}=Q_{11}\sin^{4}\theta +2\left(Q_{12}+2Q_{66}\right)\sin^{2}\theta \cos^{2}\theta +Q_{22}\cos^{4}\theta$$

$${\overset{¯}{Q}}_{16}=\left(Q_{11}-Q_{12}-2Q_{66}\right)\sin\theta \cos^{3}\theta +\left(Q_{12}-Q_{22}+2Q_{66}\right)\sin^{3}\theta \cos\theta$$

$${\overset{¯}{Q}}_{26}=\left(Q_{11}-Q_{12}-2Q_{66}\right)\sin^{3}\theta \cos\theta +\left(Q_{12}-Q_{22}+2Q_{66}\right)\sin\theta \cos^{3}\theta$$

$${\overset{¯}{Q}}_{66}=\left(Q_{11}+Q_{22}-2Q_{12}-2Q_{66}\right)\sin^{2}\theta \cos^{2}\theta +Q_{66}\left(\sin^{4}\theta +\cos^{4}\theta \right)$$

where $Q_{11}$, $Q_{22}$, $Q_{12}$, and $Q_{66}$ are defined as

(7) $$Q_{11}=\frac{E_{1}}{1-v_{12}v_{21}}$$

$$Q_{22}=\frac{E_{2}}{1-v_{12}v_{21}}$$

$$Q_{12}=\frac{v_{21}E_{2}}{1-v_{12}v_{21}}=\frac{v_{12}E_{1}}{1-v_{12}v_{21}}$$

$$Q_{66}=G_{12}$$

**Fig. 4:**
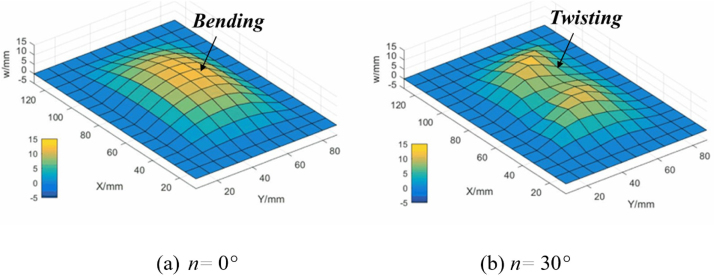
Demonstration of bending and twisting deformation for different fiber orientations.

In order to show the effect of fiber orientation on the structural deformation, Fig. 4 shows the bending deformation and twisting deformation for fiber orientation n= 0°and 30°, respectively. It notes that $E=E_{1}=E_{2}$ and $v=v_{12}=v_{21}$ for the isotropic materials.

### 3.3 FSI model

The core problem of simulating the interaction between the bubble collapse and the composite wall is to deal with the fluid–solid interface. In present work, IBM is applied to simulate the data transfer during the FSI and the detail operation of IBM is derived as follows.

The oscillation velocity $V\left(X^{n}\right)$ of the composite plate needs to be calculated according to the displacement of the composite plate [100], [101],

(8) $$V\left(X^{n}\right)=\frac{\partial \omega \left(X^{n}\right)}{\partial t}$$

where X is the spatial coordinate in the Lagrangian coordinate system. Then, the volume force $F_{T}$ in Lagrangian coordinates can be calculated according to the following formula,

(9) $$F_{T}\left(X^{n}\right)=\frac{V\left(X^{n}\right)-U\left(X^{n}\right)}{\Delta t}$$

where $U\left(X^{n}\right)$ is the corresponding Lagrangian expression of fluid velocity u in the Euler coordinate system, which can be calculated according to the following formula,

(10) $$U\left(X^{n}\right)=\underset{x^{n-1}\in g_{h}}{\sum }u\left(x^{n-1}\right)\delta_{h}\left(x^{n-1}-X^{n}\right)h^{3}$$

where $g_{h}$ is the set of Euler grid points; $\delta_{h}\left(x^{n-1}-X^{n}\right)$ is an interpolation function, and its expression is as follows

(11) $$\delta_{h}\left(x^{n-1}-X^{n}\right)=\frac{1}{h^{3}}\phi \left(\frac{x^{n-1}-X^{n}}{h}\right)\phi \left(\frac{y^{n-1}-Y^{n}}{h}\right)\phi \left(\frac{z^{n-1}-Z^{n}}{h}\right)$$

where ϕ is a discrete δ function. Here, we used the three-point smoothing function

(12) $$\phi \left(r\right)=\left\{\begin{array}{cc} \frac{55}{48}-\frac{\sqrt{3}\pi }{108}+\frac{13\left|r\right|}{12}+\frac{r^{2}}{4}+\frac{2\left|r\right|-3}{48}\sqrt{-12r^{2}+36\left|r\right|-23}\; & \\ \;+\;\frac{\sqrt{3}}{36}\arcsin\left(\frac{\sqrt{3}}{2}\left(2\left|r\right|-3\right)\right)\; & 1\leq \left|r\right|\leq 2\\ 0\; & 2\leq \left|r\right|\\ \frac{17}{48}+\frac{\sqrt{3}\pi }{108}+\frac{\left|r\right|}{4}-\frac{r^{2}}{4}+\frac{1-2\left|r\right|}{16}\sqrt{-12r^{2}+12\left|r\right|+1}\; & \\ \;-\;\frac{\sqrt{3}}{12}\arcsin\left(\frac{\sqrt{3}}{2}\left(2\left|r\right|-1\right)\right)\; & \left|r\right|\leq 1 \end{array}\right)$$

Finally, the volume force $f_{T}$ of the composite plate acting on the flow field in the Eulerian coordinate can be obtained by using the interpolation function $\delta_{h}\left(x^{n-1}-X^{n}\right)$ to deal with the volume force $F_{T}$ in the Lagrangian coordinate

(13) $$f_{T}\left(x^{n}\right)=\underset{x^{n}\in G_{n}}{\sum }F_{T}\left(x^{n}\right)\delta_{h}\left(x^{n-1}-X^{n}\right)h^{3}$$

where $G_{h}$ is the set of Lagrangian grid points. By substituting Eq. (13) into momentum equation in Eq. (3), the interaction between composite plates and bubbles can be successfully simulated

(14) $$\overset{\sim }{\rho }\left(\frac{\partial u}{\partial t}+\left(u\cdot \nabla \right)u\right)=\overset{\sim }{\rho }g-\nabla \left(p-\frac{\tau_{c}}{\chi }\nabla \cdot u\right)+\nabla \left(\mu \left(\nabla u+\nabla^{T}u\right)\right)+F_{TS}+f_{T}$$

**Fig. 5:**
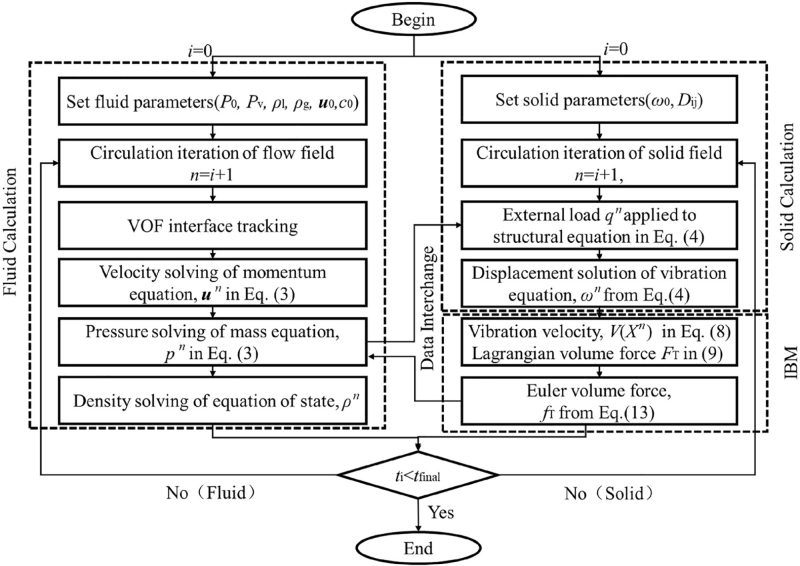
Flow chart of FSI calculation.

Fig. 5 shows the flow chart of FSI calculation. The algorithm system includes fluid, solid, and IBM calculation. The main steps are as follows: (i) calculate the fluid parameters by solving the fluid governing equation; (ii) input the pressure of fluid into the solid governing equation, and calculate displacement of solid domain; (iii) input the Euler volume force into the fluid domain by IBM; (iv) the cycle continues.

## 4 Interaction between bubble collapse and composite structure

### 4.1 The global bubble dynamics near the composite walls

Significant advances of detailed experimental bubble measurements have been made in order to obtain an understanding of bubble dynamics near the composite walls. With the advent of high-speed digital camera techniques, the visual observation [102], [103] remains a powerful tool for most types of bubble shapes. The three typical characteristics of global bubble dynamics near the various composite walls, such as bubble shape, bubble migration, and bubble collapse time, are attracted much attentions under the influence of designable properties and mechanical properties.

**Fig. 6:**
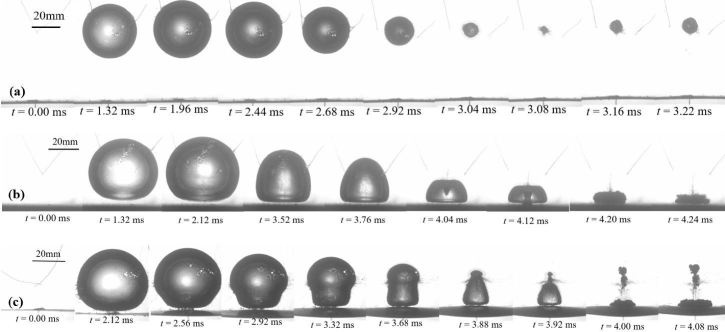
The temporal evolutions of typical bubble shapes, namely, (a) near-spherical, (b) jet-like, and (c) splitting shapes. [104].

The variations of bubble shapes are closely related to the boundary conditions. Ma et al. [104] investigated the interaction of single bubble and composite materials, focusing on the effect of variations of laminating number and fiber orientation on typical characteristics of global bubble dynamics. As shown in Fig. 6, with the changing of initial standoff distance of bubbles and the laminating number and fiber orientation of the composite walls, the bubble shapes display several patterns: near-spherical, jet-like, and splitting shapes.

**Fig. 7:**
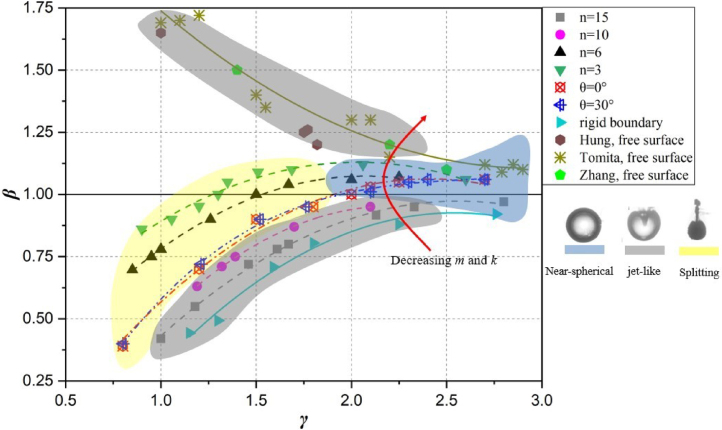
Regime map of typical bubble shapes near various composite walls.

Detailly, Fig. 7 shows the regime map of three typical bubble shapes near various composite walls. With the reduce of laminating number n, the bubble pattern transfers from the jet-like shape to the splitting shape when initial standoff γ<1.8, while it transfers from the jet-like shape to the near-spherical shape when initial standoff γ>1.8. As for the variation of fiber orientation θ, all of the bubble shapes transfers from splitting shapes to near-spherical shapes with the increase of the initial standoff, indicating that the variations of bubble shapes have less dependence on the fiber orientations of composite materials.

Regarding the bubble migration in Fig. 7, the normalized collapse position β is used to measure the bubble migration, which is defined as

(15) $$\beta =\frac{b_{RMIN}}{L}$$

where $b_{RMIN}$ is the instantaneous height above the boundary when the bubble collapses and L is the distance from the bubble center to the boundary at inception. β>1 indicates that bubbles move away from the boundaries, β= 1 indicates that bubbles undergo neutral collapse, and β<1 indicates that bubbles are attracted by the boundaries. The values of β are all clearly smaller than 1 for the bubbles near the rigid boundary and composite walls with ply number n= 10 and 15. However, this value is larger than 1 for the bubbles near the free surface investigated by Hung et al. [24], Zhang et al. [31], and Tomita et al. [55]. Interestingly, the bubbles near composite walls with n= 3, n= 6, θ= 0°and θ= 30°move in opposite directions for different γ. As shown, a bubble migrates towards the walls when γ is relatively smaller, while it is repelled from the walls when γ is larger. Furthermore, it can be found that β increases evidently with the reduce of the ply number n, while β does not change with the increase of the fiber orientation θ.

Much effort has been made to explain the physics of bubble collapse behaviors in connection with various material properties. Particularly, the physical features of the materials, such as mass m and stiffness k have great effect on bubble migration β, which causes the distinct directions of bubble migration and jet. As shown in Fig. 7, the value of m and k gradually decrease along the direction of the red arrow, which shows β increases significantly along with the decreasing of m and k. This phenomenon is also concerned by Shima et al. [53] and Tomita et al. [55].

**Fig. 8:**
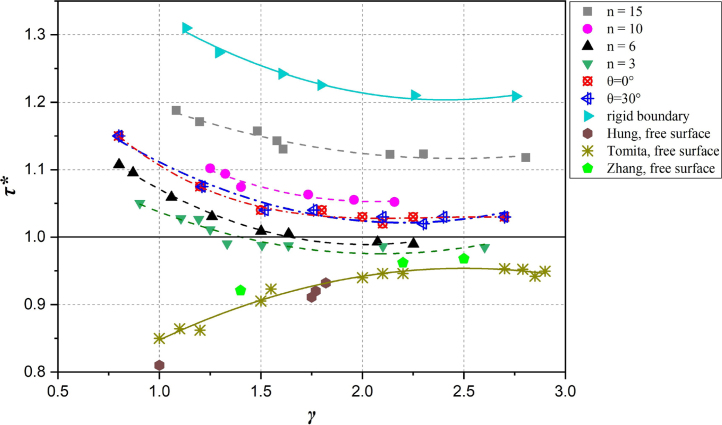
Normalized bubble collapse time against initial standoff for different composite materials.

The time of bubble collapse is other important characteristic of bubble collapse dynamics, known to vary with the boundary conditions and standoff values. To further investigate the effect of these two factors on the bubble dynamics, Fig. 8 shows the effect of the composite material properties (i.e. n and θ) on the normalized time of bubble collapse with respect to the standoff value. The normalized time of bubble collapse $\tau^{*}$ is defined as

(16) $$\tau^{*}=\frac{t_{B}}{t_{OSC}}$$

where $t_{B}$ is the time from bubble inception to minimum volume and $t_{OSC}$ is defined as the Rayleigh bubble oscillation time, reported by Rayleigh [105]. The normalized time of bubble collapse $\tau^{*}$ decreases evidently with the increase of the stand-off γ for all composite walls. For the variation of laminating number, $\tau^{*}$ increases with the increase of n. For the variation of fiber orientation, the trends of $\tau^{*}$ stay almost same for θ= 0°and 30°, indicating that the fiber orientation of composite material does not affect the variations of collapse time.

### 4.2 The tilted jets near composite walls with different fiber orientations

**Fig. 9:**
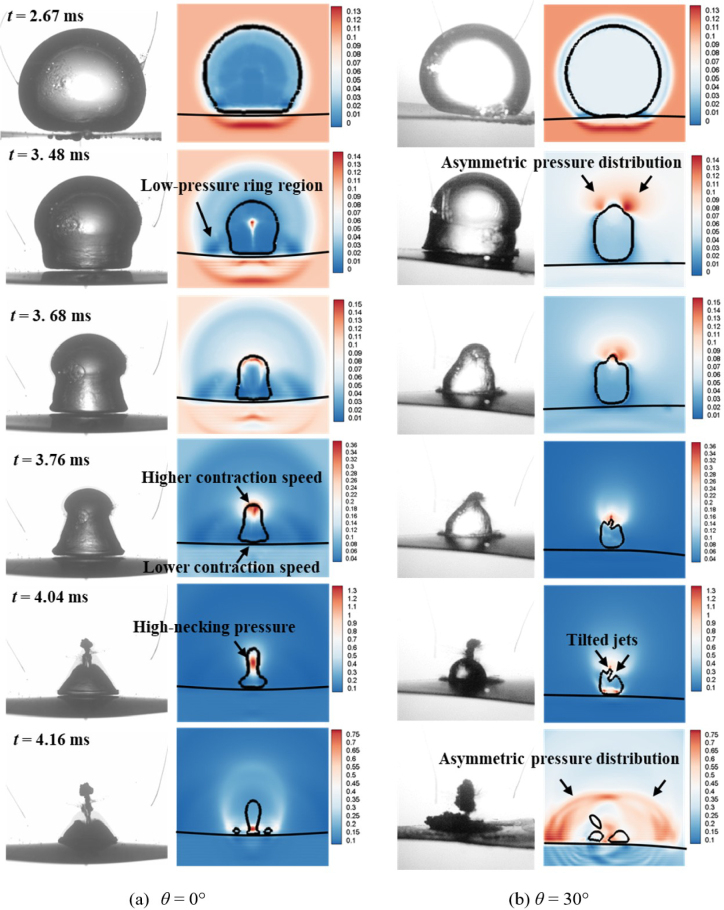
Experimental bubble shapes (left) and numerical pressure field distributions (right) near the composite walls with different fiber orientations (γ= 0.8).

In order to investigate the effect of the fiber orientations on the bubble collapse dynamics, Fig. 9 illustrates the experimental bubble shapes and numerical pressure field distributions near the composite walls with different fiber angles, namely, θ= 0°and θ= 30°. For the composite wall with θ= 0°in Fig. 9(a), the contraction velocity of bubble top wall is higher than that of bubble bottom wall, resulting in that the bubble presents the typical mushroom shape during the contraction stage (t= 2.67–3.76 ms). Furthermore, the mushroom bubble splits into two parts, annular jet and counter jet. During the collapse stage (t= 4.04 – 4.16 ms). The relevant reports can be reviewed by Brujan et al. [25], [26] and Ma et al. [106].

The formation of mushroom bubble is contributing by a low-pressure ring region appeared near the bottom of bubble. The low-pressure ring region decreases the pressure difference between inside and outside bubble, which delays the contraction process of the bottom wall of bubble, as compared to the higher contraction speed for the top wall of bubble. After that, a high-necking pressure is generated at the necking position of the bubble and pushes mushroom bubble to split into two parts: one is counter jet moving vertically upward and the other is annular jet directing vertically downward.

For the composite wall with θ= 30°in Fig. 9(b), although the bubble forms the similar mushroom shapes and splitting behaviors as compared with the case θ= 0°, the bubble shape is no longer axisymmetric, but inclined to the specific direction. During the contraction stage (t= 2.67–3.76 ms), the bottom of the bubble attaches on the surface of the composite wall, but the top of the bubble tilts to the right. During the collapse stage (t= 4.04 – 4.16 ms), the directions of the counter jet and annular jet both deviate from the center of the boundary, and the migration direction is no longer vertical just like case θ= 0°.

As observation of pressure distributions in Fig. 9(b), the formation of the inclined mushroom bubble originates from the asymmetric pressure distribution at the top of the bubble. A shear force, generated during the process of the higher-pressure side pushing bubble wall to the lower-pressure side, causes the sunken and bumped surfaces of the bubble, respectively. Then the sunken and bumped surfaces of the bubble gradually evolve into the titled counter jet and annular jet.

**Fig. 10:**
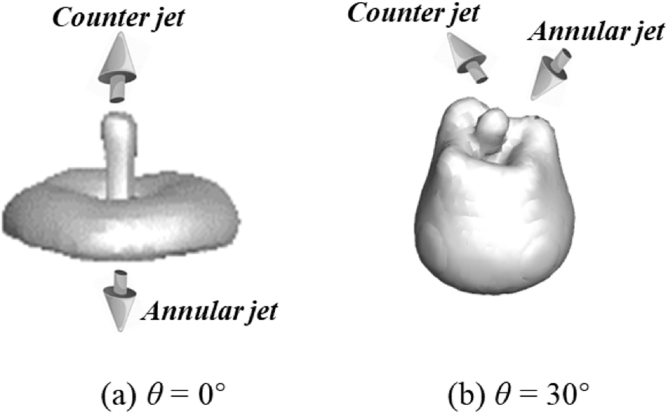
3D numerical demonstration of counter jet and annular jet (γ= 0.8): (a) θ= 0°, t= 4.16 ms; (b) θ= 30°, t= 4.04 ms.

The 3D numerical gas–liquid interfaces at the typical conditions are extracted and illustrated in Fig. 10 to show the comparisons of counter jet and annular jet near the walls with θ= 0°and θ= 30°. The bubble shapes and the formation of jets, especially for the case of θ= 30°, shows the bubble presents the asymmetric shapes. Hence, the bubble collapse dynamics near the anisotropic composite wall is a physics of three-dimensional motion, proving the numerical method should adopt the full dimension algorithm to handle this issue.

**Fig. 11:**
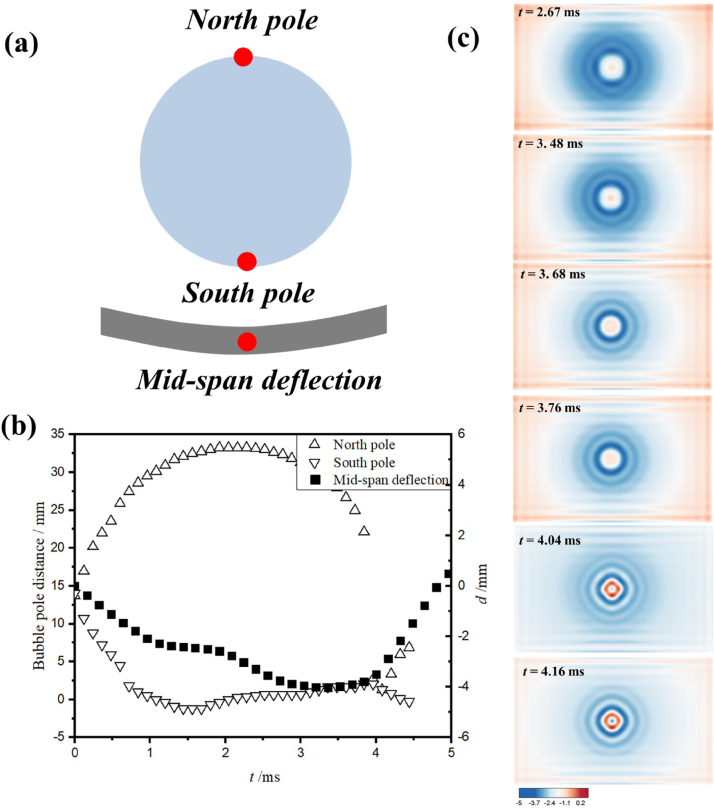
Bending deformation of composite boundary with θ= 0°along with the bubble pulsation: (a) the definition of bubble poles and the mid-span deflection of composite wall; (b) the experimental profiles of bubble poles and the mid-span deflection; (c) numerical results of composite deformation.

**Fig. 12:**
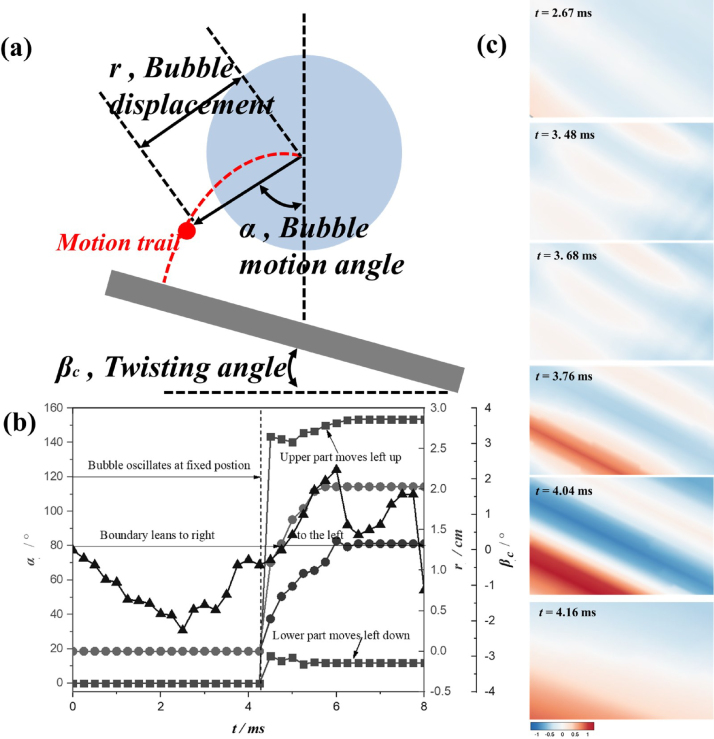
Twisting deformation of composite boundary with θ= 30°along with the bubble pulsation: (a) the definition of bubble motion (r, α) and the twisting angle of composite wall $\beta_{c}$; (b) the experimental profiles of (r, α) and $\beta_{c}$; (c) numerical results of composite deformation.

The interaction of bubble dynamics and composite wall is a typical FSI problem. Therefore, in order to further study the origins of vertical and tilted jets, respectively, Fig. 11 and Fig. 12 illustrate the structural deformations of composite walls with θ= 0°and θ= 30°along with the bubble pulsations, respectively. For the case θ= 0°in Fig. 12(a) and (b), three groups of data are experimentally monitored, namely, the north and south poles of the bubble, as well as the mid-span deflection of the composite wall. It is found that the composite wall subjected to the bubble load presents typically up and down bending movements. Furthermore, the numerical results in Fig. 12(c) shows the structural deformation of composite wall is perfectly symmetrical, which results in the formation of mushroom bubble and the vertical motion of jets.

As for the case θ= 30°in Fig. 12, due to the three-dimensional motion of the bubble, a parameter combination of (r, α) is proposed to characterize the bubble motion, where r is the displacement of bubble center and α is the angle of the bubble motion trail. Furthermore, parameter $\beta_{c}$ is defined as the twisting angle to characterize the deformation of the anisotropic wall [107]. It is found in Fig. 12(b) that the composite wall acts as typically twisting effect that the deformation of one side of composite wall is larger than that of the other side, deeply effecting and resulting in the asymmetric pressure distribution in the liquid domain firstly and then formation of crooked bubble shapes and the tilted jets. Furthermore, the numerical results in Fig. 12(c) shows the twisting visualization of the composite wall, which agree well with the trend of the experimental results.

**Fig. 13:**
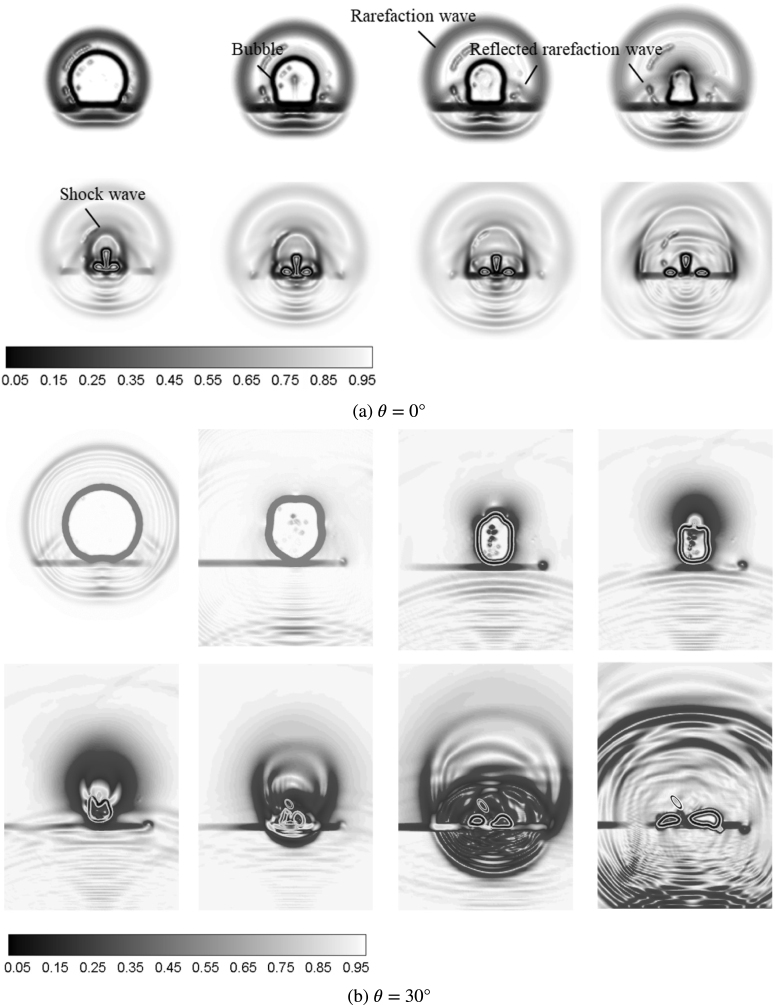
Numerical Schlieren images of bubble pressure waves near the composite boundaries with different fiber orientations.

To further study the effect of fiber orientation on the pressure characteristics of the bubbles, Fig. 13 shows the numerical Schlieren images of bubble pressure waves near the composite boundaries with different fiber orientations. In order to effectively characterize the various pressure waves released by bubble pulsation in the free field, Johnsen & Colonius [71] and Quirk & Karni [108] proposed to use numerical Schlieren image to identify the various pressure waves, in which the definition can be expressed as

(17) $$Sch=\exp\left(-C\frac{\left|\nabla \rho \right|}{\max\left|\nabla \rho \right|}\right)$$

where C= 40 for air and C= 400 for water.

As for the composite wall with θ= 0°in Fig. 13(a), the bubble firstly emits the rarefaction wave with much weak intensity during the contraction stage, and then top wall of bubble (i.e. counter jet) releases the primary shock wave with strong intensity moving in all directions during the collapse stage. Finally, the bottom wall of bubble presents the annular collapse and emits the secondary shock wave, directly impacting on the center of the composite wall.

Compared with the case θ= 0°, the case θ= 30°in Fig. 13(b) has almost same pressure wave types (such as rarefaction wave, primary and secondary shock waves) and propagation process, but the impacting centers of all pressure waves are shifted from the center of the composite wall. The direction of various pressure waves, especially for the shock wave during the collapse stage, tilts to the left part.

**Fig. 14:**
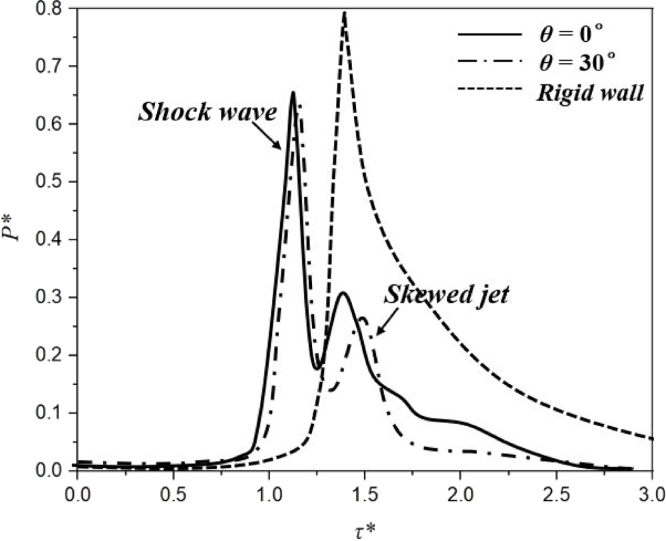
Pressure load of bubble collapse impacting on different boundaries.

The normalized pressure profiles caused by the bubble collapse in Fig. 14 are extracted at the centers of the two composite walls, as well as the rigid wall treated as control group. As observed, a largest pressure peak is found in the case of rigid boundary due to the formation of the strong high-speed jet, which has been widely investigated by Li et al. [1], [109]. Furthermore, the pressure profiles near two kinds of composite walls both present two peaks with much smaller values, resulting from the shock wave and jet. However, compared to the case of θ= 0°, the second pressure peak has much smaller value for the case of θ= 30°. This phenomenon may be caused by that the twisting deformation of anisotropic composite material results in a decrease of the pressure load acting on the monitoring point of the walls as compared with that of bending and rigid wall cases.

### 4.3 The opposite bubble migration near composite walls with different ply numbers

In order to investigate the effect of the ply number on the bubble collapse dynamics, Fig. 7 shows the experimental results of bubble migration near the composite boundaries with different ply numbers. As shown, bubbles present obviously opposite migration direction near the composite wall with different ply numbers. Bubbles are attracted towards composite wall with n= 10 and 15 for all γ conditions, while bubbles are repelled away from the composite wall with conditions (n= 3, γ>1.3) and (n= 6, γ>1.5). Therefore, the migration direction of bubbles can be controlled by designing the ply number of composite materials.

**Fig. 15:**
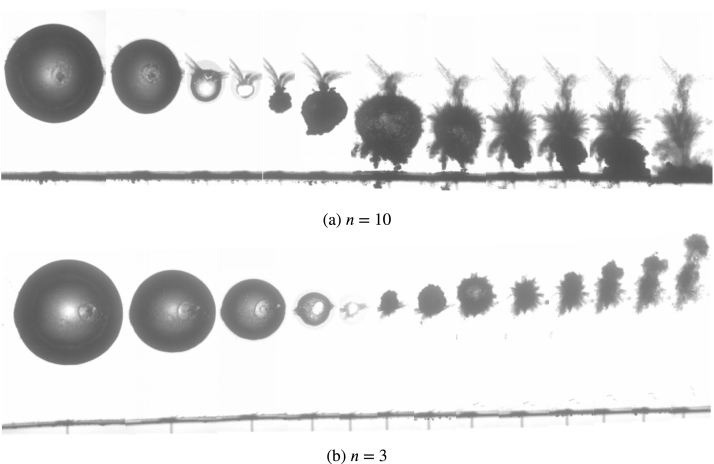
Experimental images of bubble migration near the composite boundaries with different laminating numbers (γ= 1.68). With permission of Ref. [104].

Fig. 15 shows the temporal evolution of typical bubble shapes near the composite walls with different ply numbers, namely n= 10 and 3, for same initial standoff distance of bubbles γ= 1.68. The bubbles are recorded from the maximum size to the third collapse. These experimental images show that the bubble with spherical shape is repelled from composite wall with n= 10 (γ>1 ), but migrates towards composite wall with n= 3 (β<1).

Much effort has been made to explain the physics of bubble migration towards the boundary, such as “Kelvin impulse” [110] and “mechanism of pressure gradient” [61], and so on. Particularly, the “mechanism of pulsating rarefaction wave” has attracted much attentions on revealing the formation of opposite migration of bubbles in the recent years.

**Fig. 16:**
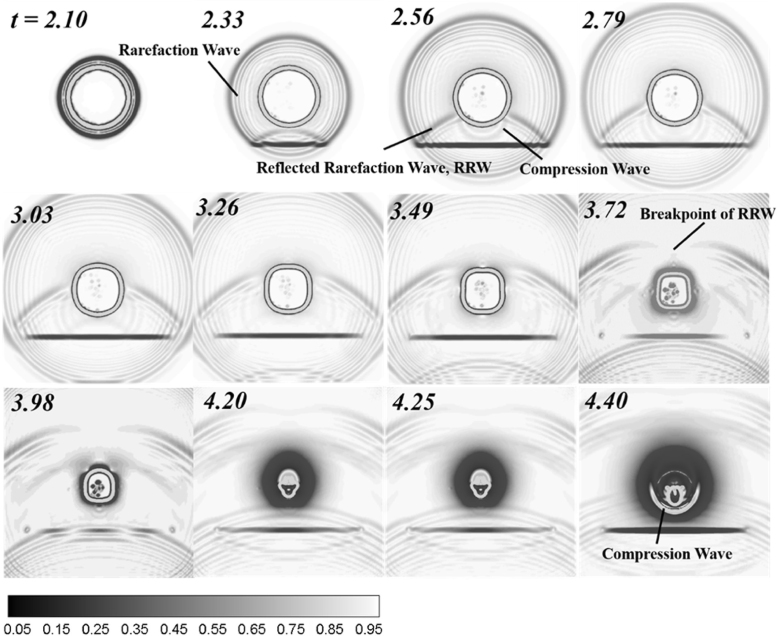
Numerical Schlieren images of bubble pressure waves near the composite wall with n= 10 [96].

The propagation and development of the rarefaction wave in the contraction stage of bubble significantly contributes to the formation of the high-speed jet and its migration. As shown in Fig. 16, the rarefaction wave radiated from the bottom of the bubble is reflected by the composite wall at t= 2.33 ms, then acts on the lower end of the bubble again at t= 2.56 ms, and transmits into the bubble interior during t= 2.79–3.03 ms. Due to the difference of gas–liquid acoustic impedance, the speed of rarefaction wave propagating in the bubble is far less than that in the liquid. Therefore, when the reflected rarefaction wave propagates to the liquid at the top of the bubble during t= 3.49–3.98 ms, the rarefaction wave inside the bubble still propagates in the bubble, so that the reflected rarefaction wave in the liquid at the top of the bubble becomes a discontinuous wave array with discontinuities. Compared to the low-pressure region behind the rarefaction wave front, the discontinuity is relatively high-pressure region due to no action of rarefaction waves [111]. The relatively high-pressure region forces the top of the bubble to sink and form a high-speed jet, which directs to the wall surface, accompanied by the formation of shock wave during t= 4.20–4.40 ms.

**Fig. 17:**
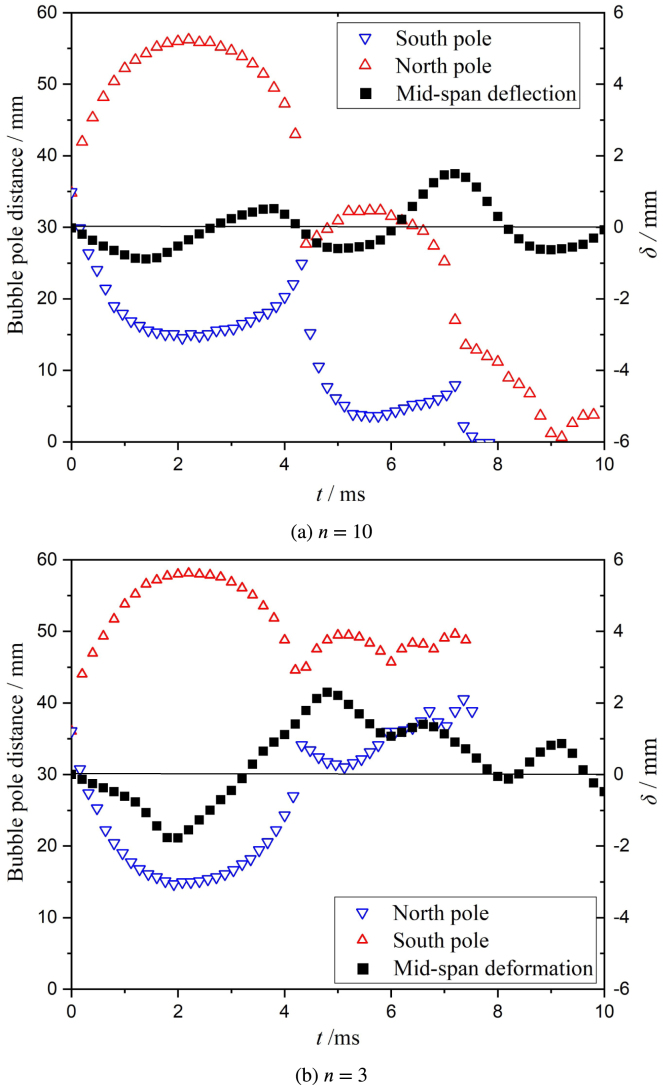
Dynamic response of composite boundaries with different laminating numbers subjected to the bubble pulsation. With permission of Ref. [104].

In order to further investigate the physical mechanism of opposite migration of bubbles, Fig. 17 shows the temporal evolution of the bubble margin and mid-span deflection corresponding to Fig. 15. It can be seen that the although composite walls have different ply numbers, they all bend downwards firstly and then spring back subsequently along the bubble pulsation. Table 6 highlights the detail differences between two cases with various n. It can be found that bubble moves downwards (β<1) when $f_{n}/f_{b}\approx$ 1.0 for n= 15 and 10, while bubble moves upwards (β>1) when $f_{n}/f_{b}\approx$ 0.5 for n= 6 and 3, which indicates two cases have opposite phase difference. It is inferred that the opposite bubble migrations are closely related to interaction of vibration phase of walls and pressure wave of bubbles. This is also confirmed by Hung et al. [24].

Compared with the case with n= 10 associated with Fig. 8, the bubble oscillating near the composite wall with n= 3 causes the opposite vibration phase difference of the wall, which may convert the reflected rarefaction wave into reflected compression wave and pushes the bubble away from the composite wall, which is same with the free surface. Because the physical characteristics of composite wall with n= 3 is much closer to the free surface as shown in Fig. 7, Fig. 8.

**Table 6:** The frequency of four walls and bubbles for the first period.

Items	Bubble	n= 15	n= 10	n= 6	n= 3
$F_{t}$/Hz	238	242	250	123	136
$F_{n-i}$/$f_{b}$	1.000	1.016	1.050	0.516	0.571
β		<1	<1	>1	>1

Four cases are all performed when γ= 1.68 and $R_{m}=$ 21 mm;

$f_{n}$ is the frequency of the composite wall vibration where n is 15, 10, 6, and 3;

$f_{b}$ is the frequency of the bubble oscillations.

### 4.4 The effect of material properties on bubble migration

In order to quantitatively characterize the effect of material properties of walls on the bubble migration, Gibson and Blake [51] proposed the normalized mass and stiffness coefficients corresponding to the bubble dynamics, which can be expressed as

(18) $$m^{*}=\frac{m}{\rho R_{m}^{3}}\;k^{*}=\frac{k}{R_{m}\left(p_{\infty }-p_{v}\right)}$$

where $R_{m}$ is the maximum radius of bubble. Hung et al. [24], Brujan et al. [25], [26], and Shima et al. [53] applied both of normalized parameters to measure the common materials, such as Al, steel, rubber and PAA, under the bubble actions. However, the two parameters can only describe the mass and stiffness characteristics of the boundary respectively, but cannot comprehensively represent the specific stiffness of the materials.

The parameter $k^{*}/m^{*}$ in present work is proposed to characterize the specific stiffness of the composite walls. In the community of material science, the specific stiffness is defined as the ratio of Young’s Modulus with the density. Due to that Young’s Modulus is proportional to stiffness and density is proportional to mass, normalized variable $k^{*}/m^{*}$ can effectively characterize the specific stiffness of the materials.

**Fig. 18:**
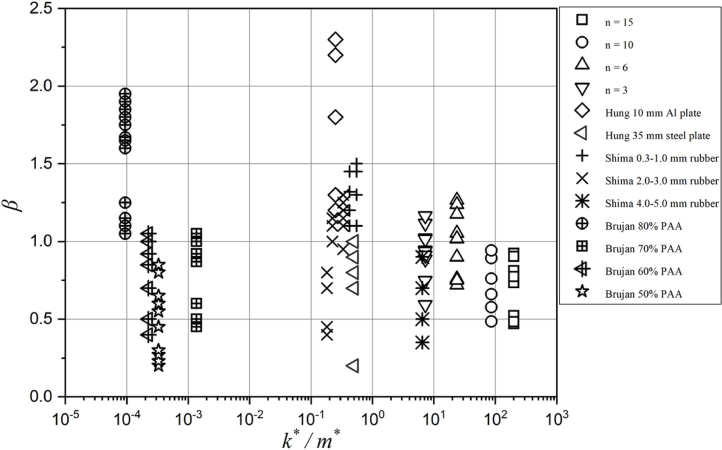
The relationship between $k^{*}/m^{*}$ and β. Data from works of Hung et al. [24], Brujan et al. [25], [26], and Shima et al. [53].

As shown in Fig. 18, the composite material series dominate the highest specific stiffness, followed by Shima’s rubber series and Hung’s metal series. And the specific stiffness from Brujan’s PAA material with different concentrations ranks bottom in the sequences. In addition to rankings of the specific stiffness, the direction of bubble motion needs to be considered comprehensively. Though the specific stiffness of the composite wall with n= 15 is higher than n= 10, 6, and 3 in turn in our composite material series, β are all smaller than 1 in the cases of n= 15 and 10, indicating bubbles all move towards the boundaries and imposes much pressure load on boundaries. Parts of data in the cases of n= 6 and 3 are β>1, showing that those composite materials cannot only have the highest specific stiffness, but also can resist bubble movement towards the boundaries, which is desirable to achieve.

To theoretically predict the direction of the bubble migration under the influence of material properties and build detail mathematic expressions, Best and Blake [112] developed a sophisticated Kelvin impulse to predict the bubble migration under the influence of material properties of various walls. The definition of Kelvin impulse I is

(19) $$I=-\frac{2\pi R_{m}^{5}{\left(6\Delta p\rho_{l}\right)}^{1/2}}{9L^{2}}\eta B\left(\frac{7}{6},\frac{3}{2}\right)e_{\xi }-\frac{4\sqrt{6}\pi R_{m}^{4}\rho_{l}^{3/2}g}{9{\left(\Delta p\right)}^{1/2}}B\left(\frac{11}{6},\frac{1}{2}\right)e_{z}$$

where $\Delta p=p_{\infty }-p_{v}$ is the pressure difference; $p_{v}$ is the saturated vapor pressure; $p_{l}$ is the density of the liquid; B (,) is the beta function; and η is a variable related to the mass m. I is proportional to β so that it can be used to predict the bubble migration near the specific boundaries. However, Kelvin impulse fails to predict the bubble migration near the flexible composite walls, because η in Eq. (19) just consider the mass m of the walls, but does not take the stiffness k into account. And it is very difficult to derive the theoretical equation including the stiffness coefficient of the walls.

In recent years, machine learning methods have attracted great attentions and provide an alternative choice to solve above problems. It is primary to build relationship between bubble collapse position and major external factors (such as the physical properties of walls, pressure, and gravitational acceleration), which can be defined as

(20) $$\beta =f\left(m,k,R_{m},L,\Delta p,\rho_{l},g\right)$$

where f is the mapping function between input and output.

**Fig. 19:**
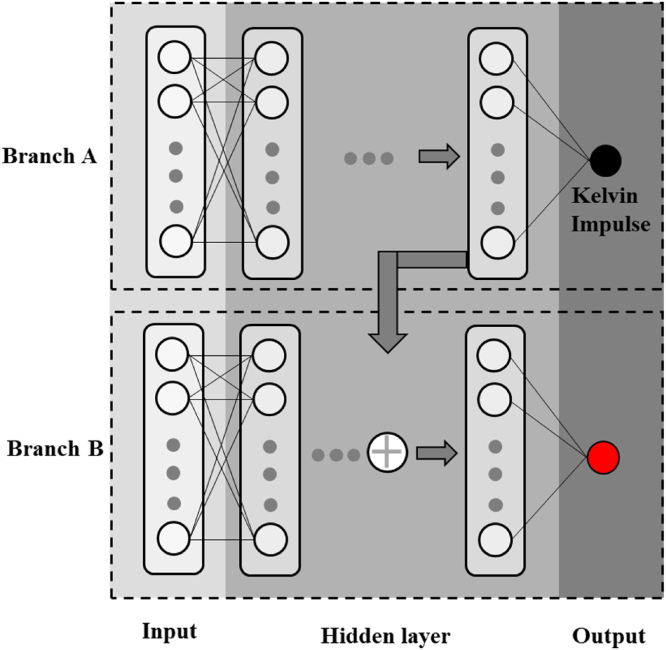
The schematic drawing of two-branch deep neural network (TBDNN) model embedded with the Kelvin impulse.

In order to improve the high-fidelity bubble migration results and further reduce dependence on the quantity of training data, Ma et al. [104] proposed to use two-branch deep neural network (TBDNN) to further improve and perfect the function of Kelvin impulse. The major external factors in Eq. (20) are divided into two groups after nondimensionalization,

(21) $$\beta =f\left(\underset{\text{Kelvin impulse}I}{\underset{︸}{\left(m^{*},\gamma ,\delta \right)}},\underset{\text{Deep Neural Network}}{\underset{︸}{\left(m^{*},\gamma ,\delta ,k^{*}\right)}}\right)$$

where $\delta =\sqrt{\frac{\rho_{l}gR_{m}}{\Delta p}}$ is the normalized gravitational acceleration. The framework of deep neural networks is established in Fig. 19 that the branch A is used to train the Kelvin impulse, and branch B is trained by the deep neural networks. The framework of TBDNN is also adopted by Ling et al. [113].

**Fig. 20:**
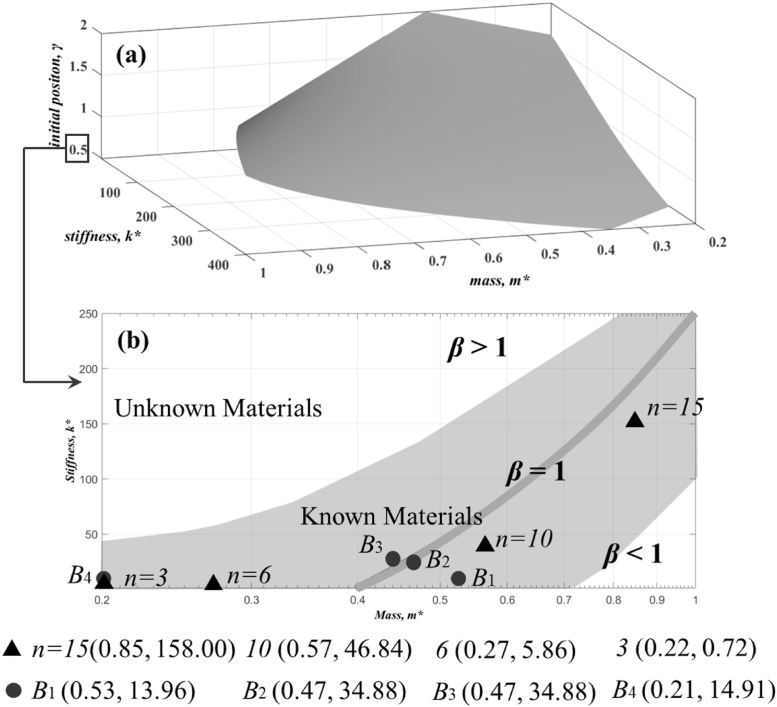
Regime map depending on $m^{*}$, $k^{*}$, γ, and β. Red dots represent the classic boundaries from Tomita et al. [52]. With permission of Ref. [104].

Fig. 20(a) shows a regime map depending on $m^{*}$, $k^{*}$, γ and β predicting by the TBDNN algorithm. Three variables $m^{*}$, $k^{*}$, γ together determine one critical surface where normalized bubble collapse position is β= 1, indicating bubble oscillation in a fixed position without motion. To show a clearer combined effect of both variables on the bubble migration, Fig. 20(b) shows a two-dimensional plot layer with coordinates of $m^{*}$ and $k^{*}$. Cases n= 10 and n= 15 scatter on the region of β>1, which means those materials will repel the bubble from the boundary. Cases n= 3 and n= 6 locate on the region of β<1, which indicates the bubbles are attracted by those materials. This plot could give guidance for the design of hydromechanical materials from the point of view of bubble motion. As known, the micro-jet and bubble migration are considered to be important mechanisms to cause the cavitation erosion of fluid machinery. Therefore, the physical properties of materials used in hydromechanics should be chosen in the region of β>1.

## 5 Future research directions

Although much progress has been made on the experimental and numerical modeling bubble dynamics near composite walls, further improvement is still necessary. Much effort should be made, especially the visual measurements of dynamic response of composite walls.

### 5.1 The macro-scale responses of composite wall subjected to bubble pulsation

The transient macro-scale responses of composite wall excited by bubble collapse load moves and changes rapidly, because the pressure load of shock wave and high-speed jet produced by bubble collapse is completed in a short time from tens of microseconds to several milliseconds. It is a great challenge to measure the transient impact inside the structure. The traditional experimental methods, such as pressure sensor and strain gauge, cannot be applied in the measurements of macro-scale responses of composite wall, due to two points:

• The composite walls usually exhibit the anisotropic characteristics, which is multi-dimensional deformation. It is contradictory to the pressure sensor and strain gauge based on single point or local measurement.
• Generally, the pressure sensor and strain gauge need to adhere to the surface of the workpiece to be tested, hence they are difficult to achieve non-contact measurements of the dynamic responses of complex structure.

**Fig. 21:**
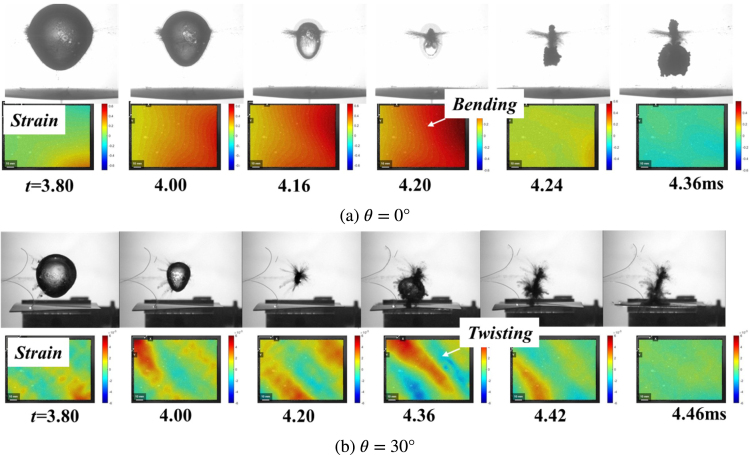
Dynamic response of composite walls based on DIC technique [96].

Digital image correlation (DIC) is a full 3D full-filled technique for deformation measurement, which is developing rapidly over the years [114]. Images are typically acquired from X-ray Computed Tomography systems, but can equally be obtained by Magnetic Resonance Imaging systems for biological subjects, or via optical tomography for transparent media. Therefore, DIC can meet the two main challenges in the macro-scale deformation measurements of composite walls subjected to the bubble pulsation, as shown in Fig. 21.

**Fig. 22:**
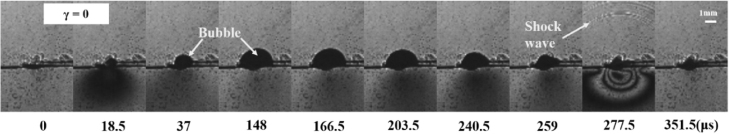
The bubble-induced stress field captured by the photoelastic imaging technique [96].

### 5.2 The micro-scale stress wave propagation induced by bubble pulsation

All of the above is based on the study of the surface of the solid and the fluids surrounding the solid, while the interior of the solid is bound to be affected as well. The velocity disturbance of structural particle changes significantly, under the action of bubble load, especially high-speed jet and shock wave with the characteristics of high instantaneity, heavy load and strong nonlinearity. The corresponding disturbances of stress and strain propagate in the form of mechanical waves, namely stress wave propagation. Solids can propagate both longitudinal and transverse waves, so the study of waves is more complex than that of fluids, but it is also an important part of the explanation of fluid–solid coupling mechanism. The research on the evolution characteristics of structural stress wave under the action of bubble load is an important foundation and premise to reveal the mechanism of structural macro dynamic response.

Photoelastic imaging technique [115] is a promising approach to achieve the fine measurement of stress waves subjected to bubble pulsation. Fig. 22 shows the visualization of stress field (or interference patterns) induced by bubble collapse based on photoelastic imaging technique. However, it still meets some challenges before application in the dynamic measurements of composite materials:

• The interference patterns in the results of photoelastic imaging are comprehensive outcome of the longitudinal wave, shear wave and surface wave. Much efforts should be made in decoupling analysis of various pressure waves with theoretical analysis method (i.e. ray-tracing analysis) and numerical simulation (i.e. finite element analysis) in the future works.
• CFRP composite materials are generally opaque, which is contradictory to fundamentals of photoelastic imaging that the fringe orders are obtained from the optical variations of the materials. Hence, studies are also primary on decolorization and hyalinization of composite materials.

## 6 Concluding remarks

In summary, recent progress on bubble dynamics near composite walls are reviewed in present work. A throughout survey of experimental and numerical methods for bubble behaviors near various boundaries are presented to reveal the interaction mechanisms of bubble dynamics and composite walls. Meanwhile, the limitations in the visual measurements of dynamic response of composite walls are still existed and more work is still needed.

In the present article, three topics are mainly involved.

(1) The progress made in the experimental and numerical modeling and approaches are summarized for bubble dynamics near various composite walls. In experiments, synchronous acquisition system for the collection of bubble shapes and dynamic response of boundary are developed, with focusing on the bubble generator of low-voltage discharge and designability of composite materials. In numerical method, the FSI algorithm of bubble dynamics and composite wall interaction is developed, including the modified N–S equation with considering both strong and weak compressibility, vibration equation of anisotropic material, and the immersed boundary method of achieving the data transfer in the calculation process of Euler grid and Lagrange grid.
(2) The effect of designability of the composite materials on the bubble dynamics is discussed. The anisotropic composite walls resulted from the variations of fiber orientations exhibit the bending and twisting deformation, contributing to the formation of symmetrical and tilted jets. Furthermore, reflected rarefaction waves during the contraction stage of bubbles is an important physical mechanism to result in the bubble migration. And the bubble migration can be controlled by the variations of ply number of composite materials, which is closely related to the propagation of reflected rarefaction waves.
(3) Specific stiffness of composite material has a significant effect on the bubble migration, enhancing the critical condition of neutral collapse position. A modified deep neural network method embedded with Kelvin impulse is proposed to predict the effect of material properties on the bubble migration, and corresponding results show the composite material with higher specific stiffness on the premise of rebounding bubbles away, as compared with the commonly used materials.

## CRediT authorship contribution statement

**Yichen Zhu:** Writing – review & editing, Writing – original draft, Investigation. **Xiaojian Ma:** Writing – original draft, Investigation, Conceptualization. **Ruiquan Zhou:** Investigation. **Yuwei Sun:** Investigation, Conceptualization. **Mindi Zhang:** Supervision, Project administration.

## Declaration of competing interest

The authors declare that they have no known competing financial interests or personal relationships that could have appeared to influence the work reported in this paper.
